# Development of Bioepoxy Resin Microencapsulated Ammonium-Polyphosphate for Flame Retardancy of Polylactic Acid

**DOI:** 10.3390/molecules24224123

**Published:** 2019-11-14

**Authors:** Kata Decsov, Katalin Bocz, Beáta Szolnoki, Serge Bourbigot, Gaëlle Fontaine, Dániel Vadas, György Marosi

**Affiliations:** 1 Department of Organic Chemistry and Technology, Budapest University of Technology and Economics, H-1111 Budapest, Budafoki street 8., Hungary; decsovkata@oct.bme.hu (K.D.); bszolnoki@mail.bme.hu (B.S.); dvadas@oct.bme.hu (D.V.); gmarosi@mail.bme.hu (G.M.); 2 Unité Matériaux et Transformations (UMET), CNRS UMR 8207, École Nationale supérieure de Chimie de Lille, University of Lille, Bâtiment C6, Cité Scientifique, 59652 Villeneuve d’Ascq Cedex, France; serge.bourbigot@ensc-lille.fr (S.B.); gaelle.fontaine@ensc-lille.fr (G.F.)

**Keywords:** polylactic acid, intumescent flame retardant, ammonium polyphosphate, microencapsulation, bioepoxy

## Abstract

Ammonium-polyphosphate (APP) was modified by microencapsulation with a bio-based sorbitol polyglycidyl ether (SPE)-type epoxy resin and used as a flame retardant additive in polylactic acid (PLA) matrix. The bioresin-encapsulated APP (MCAPP) particles were characterized using Fourier transform infrared (FTIR) spectroscopy and Raman mapping, particle size distribution was determined by processing of scanning electron microscopic (SEM) images. Interaction between the APP core and the bioresin shell was revealed by combined thermogravimetric analysis (TGA)‑FTIR spectroscopy. The APP to SPE mass ratio of 10 to 2 was found to be optimal in terms of thermal, flammability, and mechanical properties of 15 wt% additive containing biocomposites. The bioresin shell effectively promotes the charring of the APP-loaded PLA composites, as found using TGA and cone calorimetry, and eliminates the flammable dripping of the specimens during the UL-94 vertical burning tests. Thus, the V-0 rating, the increased limiting oxygen index, and the 20% reduced peak of the heat release rate was reached compared to the effects of neat APP. Furthermore, better interfacial interaction of the MCAPP with PLA was indicated by differential scanning calorimetry and SEM observation. The stiff interphase resulted in increased modulus of these composites. Besides, microencapsulation provided improved water resistance to the flame retardant biopolymer system.

## 1. Introduction

The growing environmental awareness of society and the shortage of petroleum-based resources has led to extensive research in developing bio-based plastics as a means of solving the disposal problem and reducing the environmental impact of the non-degradable petrochemical-based plastics [1,2,3,4,5].

Among biodegradable polymers, polylactic acid (PLA) has received an increasing amount of attention because of its controllable biodegradability and abundant renewable source (lactic acid can be efficiently produced by fermentation from renewable resources [6]). Moreover, its mechanical properties, processability at high melting temperature, and transparency are excellent [7]. PLA is used not only for packaging and medical devices [8,9,10,11,12], but it is also applicable for producing long life-cycle goods, such as in automotive components, the electrical industry, building materials, and the aerospace industry due to ecological and economic advantages [13,14,15,16]. However, while these latter mentioned durable engineering products require a remarkable flame retardant grade, PLA is inherently flammable because of its molecular structure. Without flame retardants (FRs), it cannot meet the safety standards. This necessitates the continuous development of widely applicable, highly effective FRs for the modification of PLA [17,18,19,20]. Intumescent flame retardants (IFRs), consisting of acid source, a carbonization agent, and a blowing agent [21], are considered promising for this purpose, owing to their low smoke emission, low toxicity, and low corrosion [22,23,24,25,26]. Upon heating, fire retardant intumescent materials form foamed cellular charred layers on their surface, acting as a physical barrier, which can insulate the heat transfer and prevent the diffusion of the oxygen and the volatile products efficiently between gas and condensed phases [27,28,29,30,31]. In such systems, ammonium polyphosphate (APP) can act both as the acid source and the blowing agent, while pentaerythritol (PER) is most frequently used as the carbonization agent [32]. However, both APP and PER have poor compatibility with polymer matrices and they are moisture-sensitive, which often results in the reduction of flame retardancy and mechanical properties of the composites [33,34].

The microencapsulation process is one of the most effective methods to modify the surface properties of a filler through forming an outer shell to modify the interface interaction, also, it possibly results in the enhancement of flame retardancy, mechanical properties, and water resistance of some polymer composites [12,35,36,37,38]. Chen and Jiao [39] co-microencapsulated APP and PER using a hydroxyl silicone oil by in situ polymerization method. Increased limiting oxygen index (LOI) values and UL-94 vertical burning test ratings, improved water resistance, and higher thermal stability were found in the case of the microcapsule-containing PP composites compared to the composites with neat additives. The heat release rate (HRR) and mass loss rate (MLR) values obtained from cone calorimeter tests also decreased as a result of the microencapsulation.

Melamine is also often combined with APP, even in PLA matrix. Wang et al. microencapsulated APP with a melamine-formaldehyde (MF) resin to produce an intumescent flame retarded PLA biocomposite. In this system, microencapsulated ammonium polyphosphate (MCAPP) is used as the acid source, melamine (MA) acts as the blowing agent, while starch functions as the charring agent. With 30 wt% IFR, UL-94 V-0 rating and a high LOI value of 41% were reached [26].

Yang et al. [40] combined MF resin microencapsulated APP with PER. A beneficial effect was evidenced on the intumescent char formation and also, an enhanced anti-dripping effect was found, thus flame retardant PP composites passed the V-0 rating with an LOI of 23.4 wt%.

Cyclodextrin microencapsulated ammonium polyphosphate was prepared by Wang et al. [41] with the goals of improving the water durability of APP, preparing a novel functional flame retardant and improving the compatibility with ethylene-vinyl acetate copolymer (EVA), 40 wt% flame retardant was required to achieve the V-0 rating in the case of the vertical burning test.

Shi et al. produced flame retardant PLA composites with resorcinol bis(diphenyl phosphate) (RDP)-coated APP (C-APP), and RDP-coated distiller’s dried grains with solubles (C-DDGS) were also prepared and required 15 wt% C-DDGS and 15 wt% C-APP for the biocomposites to pass the UL-94 V-0 rating and reach the LOI value of 32.0% [42].

Ran et al. prepared APP microencapsulated with polysiloxane (Si) and polyborosiloxane (BSi), and introduced into PLA to improve the flame retardancy, water-resistance, and mechanical properties of flame retarded PLA composite. The flammability evaluation for the PLA with 5% BSi microcapsuled APP (BSi-APP) samples revealed that the LOI value increased to 26.7%, the UL-94 grade reached up to V-0, and the peak heat release rate (HRR) was decreased significantly. The microencapsulation of APP also improved the compatibility of APP with PLA, resulting in better mechanical properties than the control APP-contained composites under the same loading. The water-resistance of the PLA composite was also significantly improved by the introduction of microencapsulated APP [43].

In this work, a new type of microencapsulated APP was designed and prepared through in-situ polymerization. A sorbitol polyglycidyl ether-based epoxy resin shell was prepared for APP not only to improve its compatibility with PLA but also, as a biobased carbonization agent, to enhance the flame retardant efficacy of the IFR system. The effect of the thickness of the bioresin shell of APP was investigated on the thermal properties, flame retardancy, mechanical performance, and water-resistance of flame retarded PLA composites.

## 2. Results and Discussion

### 2.1. Characterization of the Microcapsules

#### 2.1.1. Fourier Transform Infrared (FTIR) Spectroscopy

**Figure 1:**
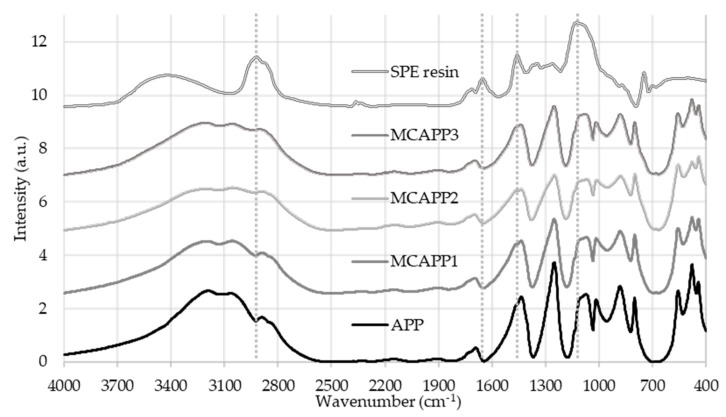
Fourier transform infrared (FTIR) absorbance spectra of the microcapsules and components.

FTIR absorbance spectra (Figure 1) taken from the microcapsules confirmed that the resin was present on the surface of the filtered and dried solid APP particles after the encapsulation procedure.

Resin-specific bands, such as 2918 cm^−1^ CH_2_-stretching, 1460 cm^−1^ CH_2_ bending, 1126 cm^−1^ C-O ether bond, and 746 cm^−1^ CH_2_ rockings appear most intensively in the spectrum of the MCAPP3 sample with the thickest bioepoxy shell.

#### 2.1.2. Raman Spectroscopy

**Figure 2:**
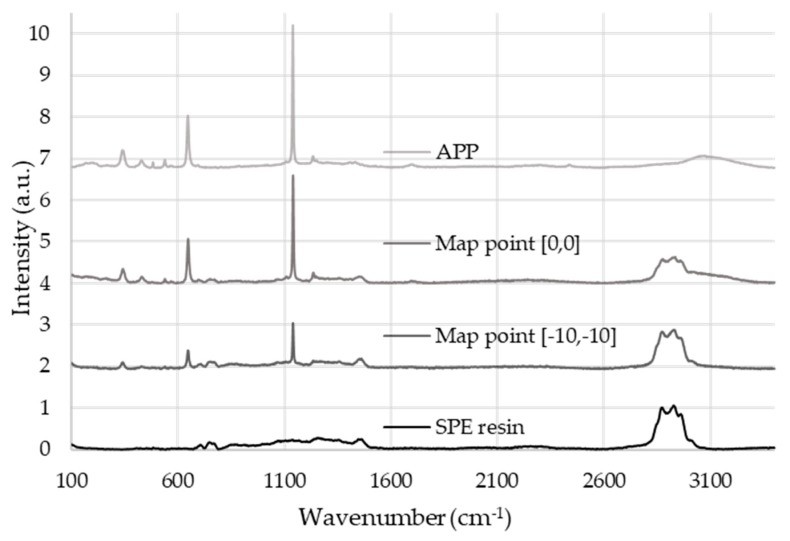
Reference Raman spectra collected from the pure components and two spectra collected from different locations of the microencapsulated ammonium polyphosphate (MCAPP3) additive.

The bioresin coating on the APP particles after encapsulation was examined by Raman mapping. For this purpose, reference spectra were taken from the starting materials, from the neat APP and the cured bioresin. Figure 2 shows the obtained reference spectra together with two Raman spectra taken from two different locations from the surface of an MCAPP3 particle with the thickest bioresin shell.

It can be observed that while APP has intense peaks at 1140 and 647 cm^−1^ assigned to the (PO_4_)^3−^ symmetric stretching vibrations and the (PO_4_)^3−^ ν_4_ bending, the resin exhibits Raman activity between 2870 and 2956 cm^−1^ (corresponding to C-H stretching vibrations; symmetric CH_2_ and CH_3_ and asymmetric CH_2_ stretch, respectively). Such significantly differing spectra makes the calculation of the spectral concentration at each point of a Raman map simple and accurate.

**Figure 3:**
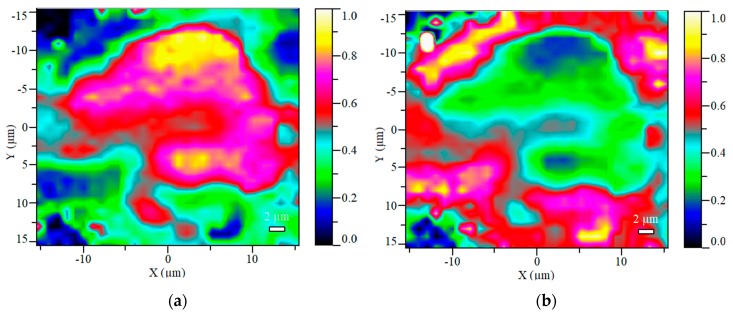
The visualized score maps of the distribution of the two major components in the MCAPP3 microcapsule (at 100× magnification): (**a**) the calculated APP content, (**b**) the calculated resin content.

The Raman maps taken from the microcapsuled additives contribute to the visualization of the core-shell structure of the prepared bioresin-encapsulated APP particles. Figure 3 shows the maps of the calculated Raman scores (i.e., spectral concentrations) obtained from an MCAPP3 particle, which visualizes the compositions calculated at each point with a two-way image.

It can be seen that the APP is mainly detected in the inside of the particle, while a large amount of resin is present on the outside of it. Although in the inner parts of particle the APP content was measured to be close to 90% of the calculated composition, the resin was detectable at every location of the surface, proving that the APP was completely covered with the resin.

#### 2.1.3. Scanning Electron Microscopy (SEM)

**Figure 4:**
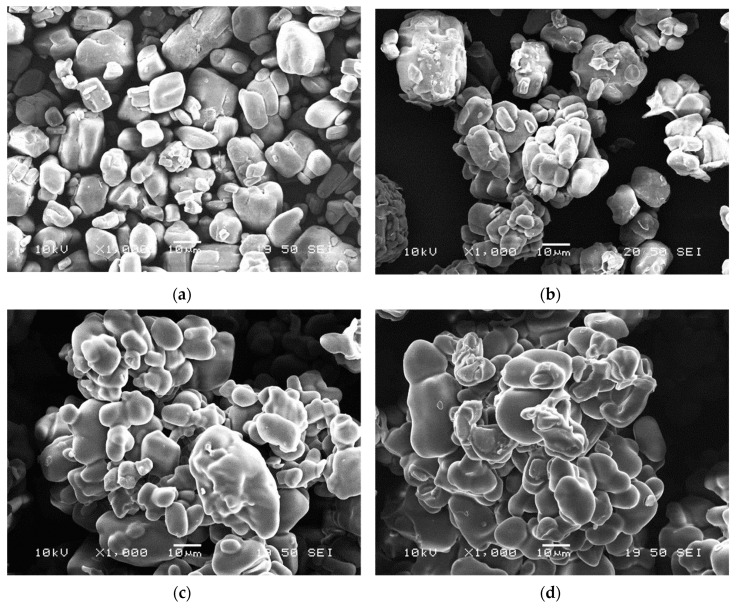
Scanning electron microscopic (SEM) images of microcapsules of different composition with 1000× magnification: (**a**) APP, (**b**) MCAPP1, (**c**) MCAPP2, and (**d**) MCAPP3.

The SEM micrographs taken from the neat and encapsulated APP particles (shown in Figure 4) demonstrate that neat APP has a blocky structure with a rough surface, while with thickening bioepoxy shell layer the surface becomes smoother and the particles are more spheroidal. Nevertheless, it can be observed that with the increasing amount of bioresin added to the solution, the particles increasingly tend to stick together (see Figure 4d) and form aggregates, which may reduce their dispersibility in the polymer matrix.

#### 2.1.4. Determination of the Particle Size and Distribution

SEM micrographs with identical 500× magnification obtained from the microcapsules and neat APP particles were analyzed. The average values of d_EC_ (diameter of a circle with an equivalent area of the measured particles) and d_M_ (maximum diameter (caliper), the maximum distance within the boundaries of a particle), calculated by the used software, are reported in Table 1 with the relative standard deviation value.

**Figure 5:**
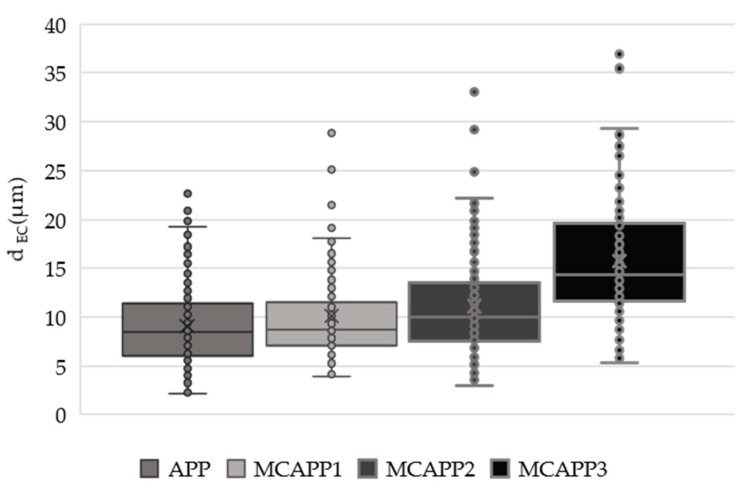
Box whiskers plot of the particles measured the diameter of a circle of equal projection area.

For a better visibility, Figure 5 shows the d_EC_ values plotted with a box plot. From these data, it can be concluded that the coating layers of MCAPP1 and MCAPP2 are around 1 µm and 2 µm, respectively. It can also be seen that the MCAPP3 sample shows a steep increase in the particle size distribution. This also suggests that particles with thicker bioresin shell layers are prone to form aggregates.

#### 2.1.5. Thermogravimetric Analysis (TGA)

**Figure 6:**
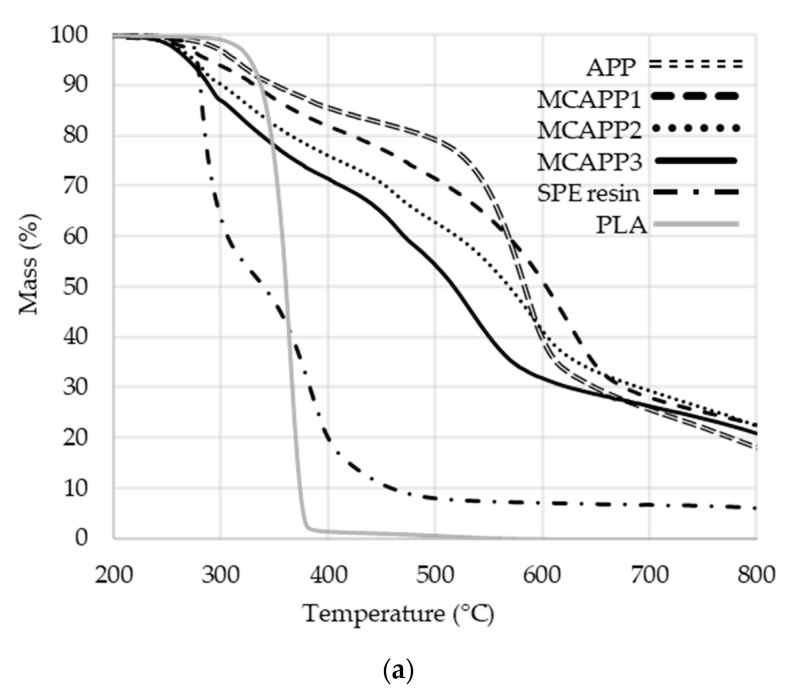

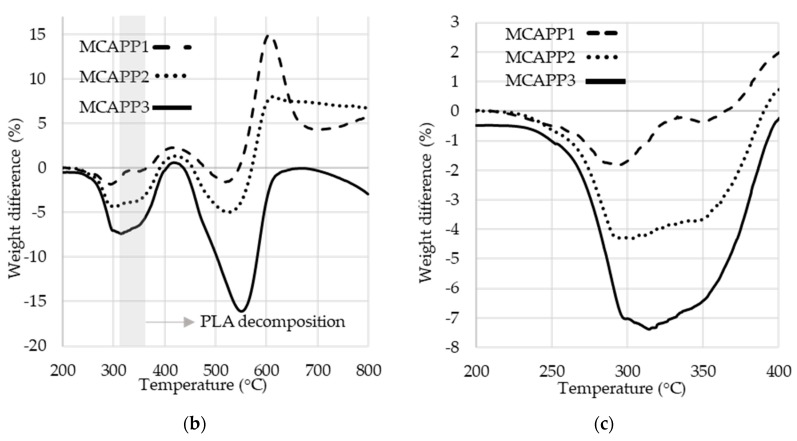
(**a**) Thermogravimetric analysis (TGA) curves of polylactic acid (PLA), APP and MCAPP additives as measured under N_2_ atmosphere with the heating ramp of 10 °C/min. (**b**) Difference between the measured and the theoretical TGA curves. (**c**) Difference between the measured and the theoretical TGA curves in the temperature range of 200–400 °C.

According to the TGA results, with increasing resin ratio, the initial decomposition temperature of the microencapsulated APP shifted to lower temperatures (see Figure 6a). This decrease of decomposition temperature showed a greater tendency than expected on the basis of the composition of the capsules.

In the case of the MCAPP2 (representing SPE to APP mass ratio of 2:10) sample, the onset of decomposition is approximately at the midpoint of those of the two pure starting materials; therefore the difference from the expected decomposition properties was further investigated. It was assumed that the composition of the capsule was close to the concentration of the substances used during the microencapsulation process, i.e., 11.76, 21.10, and 28.57 wt% bioresin content. Using these ratios, theoretical decay curves were obtained by summing the TGA curves of the starting materials according to the calculated composition percentages (e.g., the theoretical TGA curve of the MCAPP1 is composed of 88.24% of the APP curve and 11.76% of the crosslinked resin curve) and compared to the measured TGA curves. For comparison, the theoretical curves were subtracted from the real curves. The resultant comparative curves are shown in Figure 6b.

The most important range of the comparative curves is between 200 and 400 °C (see Figure 6c) since the thermal decomposition of the PLA starts around 325 °C. While in the case of the MCAPP1 sample, only a slight difference between the theoretical and measured curves can be observed, with increasing the amount of the resin, the degradation rate of the coated particles is higher at a given temperature than expected from the theoretical curves. This phenomenon can be explained by the interaction between APP and the bioepoxy resin.

#### 2.1.6. Thermogravimetry-FTIR Spectrometry

**Figure 7:**
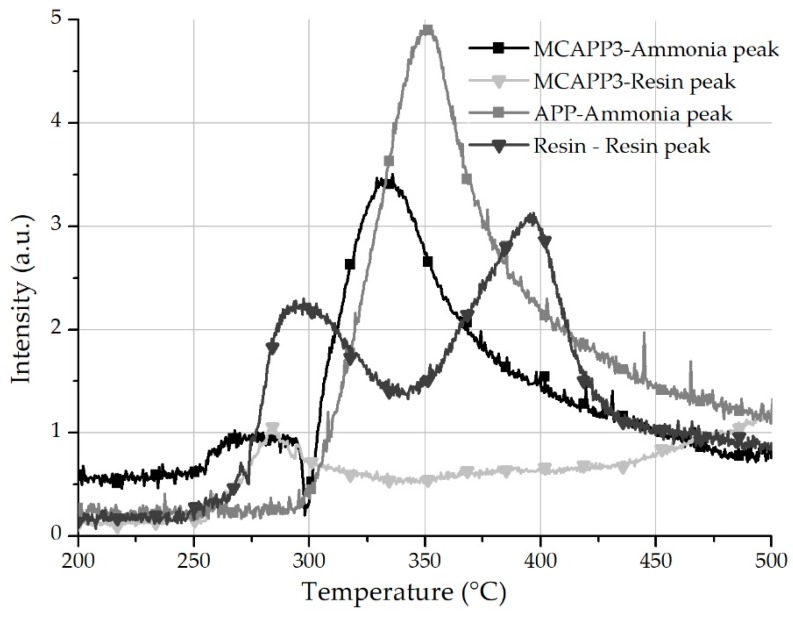
The trace of the chosen peaks of ammonia and resin degradation product in absorbance units versus the temperature during thermogravimetry-Fourier transform infrared (TG-FTIR) analysis.

In order to further investigate the decomposition process of the MCAPPs and the potential APP–bioresin interaction, TG-FTIR tests were carried out. Measurements were performed on three types of samples: untreated APP, cryo-ground cross-linked bio-resin, and MCAPP3 microcapsule. The time course of the spectra was observed in three-dimensional (3D) graphs. In the lower temperature range, two characteristic peaks appear in the FTIR spectrum at 3150 and 3050 cm^−1^ wavelengths, corresponding to amine functional groups from the decomposition of the bioresin. The decomposition of neat APP is associated with the appearance of the two intense peaks of ammonia at 966 and 931 cm^−1^. In Figure 7, the intensities of these typical wavelength values of the degradation products were plotted against temperature. It can be observed that in the case of the MCAPP3 particles, after the appearance of the resin’s degradation products, the decomposition of APP is more intense and reaches its maximal rate at a lower temperature than that of untreated APP. This faster degradation occurs at around 320 °C, just like the anomaly observed between the measured and theoretical TGA curves (Figure 6c). It is assumed that the decomposition of APP is facilitated by the degradation products of the bioresin. This effect can be beneficial in terms of flame retardant efficacy because the formation of the foamed protective layer begins closer to the decomposition temperature of polylactic acid, thus keeping more of its decomposition products in the solid phase.

### 2.2. Characterization of Flame Retarded Polylactic Acid (PLA) Composites

All the test methods were performed on PLA composites with 15 wt% loading of neat or microencapsulated APP, meaning that the MCAPP-containing samples have lower phosphorus content than the neat APP-containing composite. Where applicable, two types of neat PLA were examined to study the effect of the processing method on the polymer. PLA_GRAN was hot-pressed from dried PLA granulates while PLA_MIX was first kneaded in the internal mixer and then hot-pressed.

#### 2.2.1. Scanning Electron Microscopy (SEM)

**Figure 8:**
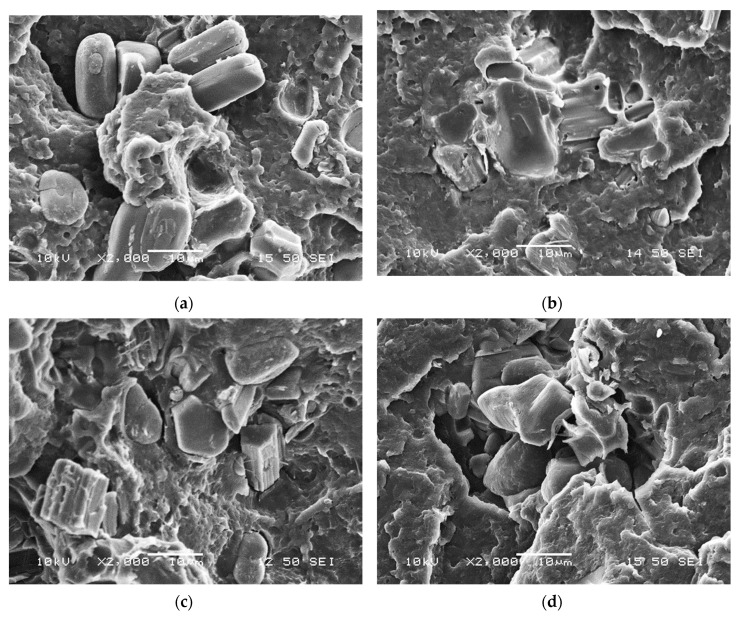
Scanning electron microscopic images of fracture surfaces with 2000× magnification: (**a**) PLA + APP, (**b**) PLA + MCAPP1, (**c**) PLA + MCAPP2, and (**d**) PLA + MCAPP3.

SEM images were taken from the cryo-fractured surfaces of the flame retarded PLA composites to investigate the distribution of the additives within the matrix and also their interfacial interaction. In Figure 8a, clear detached boundaries can be seen between the neat APP particles and PLA, the particles are pulled out of the matrix indicating poor compatibility between them.

On the contrary, the PLA + MCAPP1 (Figure 8b) and PLA + MCAPP2 (Figure 8c) samples show less separated particles and unsharp phase boundaries, indicating better interfacial interaction of the bioepoxy encapsulated APPs with PLA. In Figure 8d, agglomeration of the MCAPP3 particles can be observed, the APP particles stuck by the bioepoxy resin capsules could not be dispersed perfectly during thermomechanical preparation of the sample. The inhomogeneous distribution of the MCAPP3 particles might affect the flammability and mechanical properties of the PLA composites.

#### 2.2.2. Gel Permeation Chromatography (GPC)

**Figure 9:**
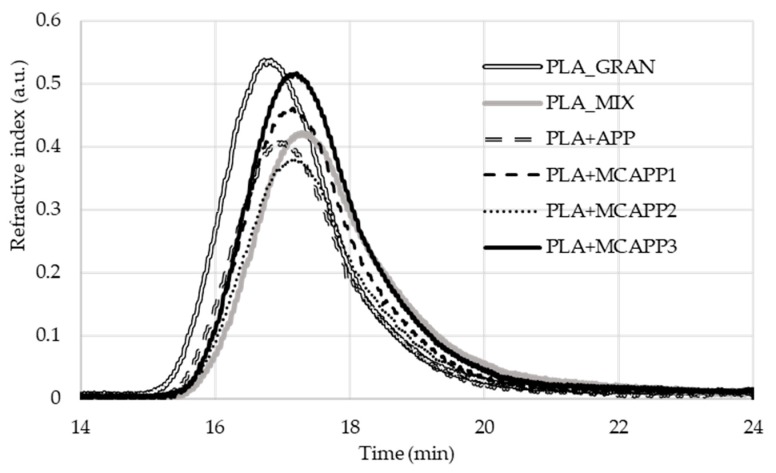
The gel permeation chromatography (GPC) chromatogram of the virgin, unprocessed PLA and the prepared polymer samples.

In order to preserve the mechanical properties of PLA, it is very important to avoid noticeable degradation, i.e., a significant reduction of the molecular weight during the processing steps. To investigate this effect, gel permeation chromatography (GPC) was performed on the samples. The obtained refractive index chromatograms can be seen in Figure 9.

**Figure 10:**
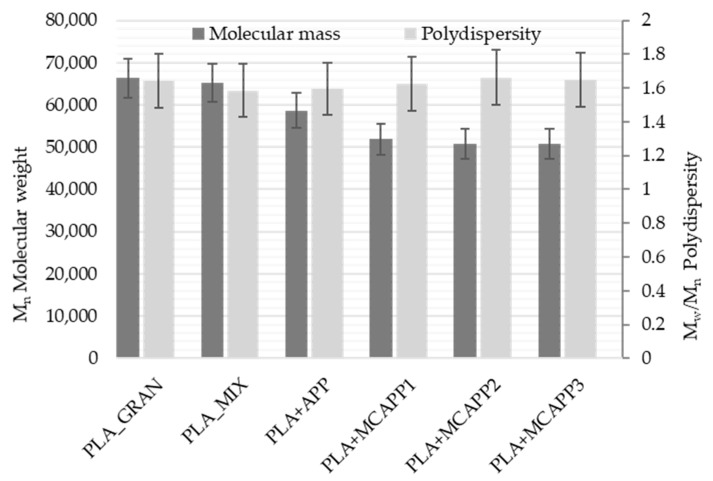
The number average molecular weight (left columns) and the polydispersity (right columns) rate of the biopolymer samples.

The number-average molecular weight (M_n_) thus obtained from the analysis of the chromatograms is illustrated in Figure 10. The degree of polydispersity, which was calculated by the quotient of the weight-average molecular (M_w_) weight and the number-average molecular weight (M_n_), is also presented in Figure 10.

According to the official datasheet, the used PLA grade has a number-average molecular weight of 68,000 g/mol, which was verified with good approximation by our measurement. The GPC results show that the molecular weight loss of the MCAPP-containing samples is larger than that of the additive-free or neat APP-containing PLA, which can possibly affect its physical properties. This molecular weight loss can be caused by acid-catalyzed degradation during processing.

#### 2.2.3. Differential Scanning Calorimetry (DSC)

The thermal properties of the flame retarded PLA composites were examined by performing heating/cooling/heating differential scanning calorimetry (DSC) runs (Table 2). From the first heating, fairly high crystalline content ranging between 45%–51% was obtained for all the examined samples, indicating that under the hot-pressing conditions, highly crystallized solid structures were formed. Such high relative crystalline contents are generally associated with increased moduli. During cooling from the melt, the additive-free PLA_MIX experienced noticeably less crystallisation than the flame retardant containing composites. The polymer/filler interface can play the role of a nucleator facilitating crystallization during cooling. This additive induced crystallization is more emphasized when MCAPPs were added. The increase of crystallization percentage during cooling is associated with the increased total crystalline percentage obtained after the second heating run. It can also be seen that the cooling crystallization peak shifts to higher temperatures in the case of MCAPP-containing PLA composites, which indicate increasing nucleation efficiency of the PLA/bioresin interface compared to the PLA/APP interface. Based on this, the APP particles with bioresin shell are assumed to have better interfacial bonding to PLA than the neat APP, as also suggested based on the SEM observation (Figure 8).

#### 2.2.4. Thermal Degradation

**Figure 11:**
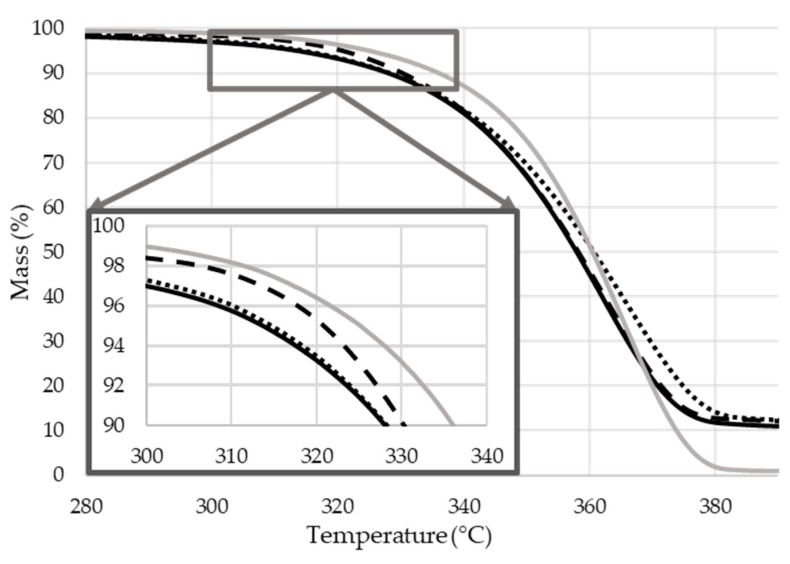
The thermogravimetric curves of the biopolymer composites (heating rate 10 °C/min, N_2_ atmosphere).

The thermal stability and degradation of the samples were investigated under N_2_ atmosphere. For better visibility, the thermal gravimetric curves of the flame retarded composites can be seen in Figure 11, while the gained data of all the samples are given in Table 3.

The degradation of all the samples occurs in one step. When comparing the TGA curves of the kneaded PLA sample (PLA_MIX) with the original pellet (PLA_GRAN), the decrease of the weight loss rate can be ascribed to the molecular weight reduction that occurred during the thermo-mechanical processing of the polymer.

Compared with the reference kneaded PLA (PLA_MIX), T_max_ and char residue values of all the flame retarded PLA composites increased while maximum weight loss rates decreased as a result of intumescent char formation. This char layer can act as a thermal barrier and inhibits the transition of heat and flammable gases. It can also be seen that T_5%_ of all the MCAPP-containing PLA composites are lower than that of the PLA composite with neat APP (PLA + APP), which is due to the earlier decomposition and possible interactions between APP and the epoxy resin (as also observed based on Figure 6c and Figure 7) while releasing gas and transforming to foamed char.

Among the examined flame retardants, the lowest weight loss rate and the highest amount of char, i.e., the best thermal stabilizing effect, were obtained with the MCAPP3 additive (in spite of its somewhat lower dispersibility), which represents 10.7 wt% APP and 4.3 wt% bioresin content in the PLA composite. These results indicate that the epoxy resin shell on the APP particle can effectively facilitate the formation of the char layer in the high-temperature range, which can delay the thermal degradation of PLA and protect PLA from further decomposition during a fire.

#### 2.2.5. Water-Resistance Tests

**Figure 12:**
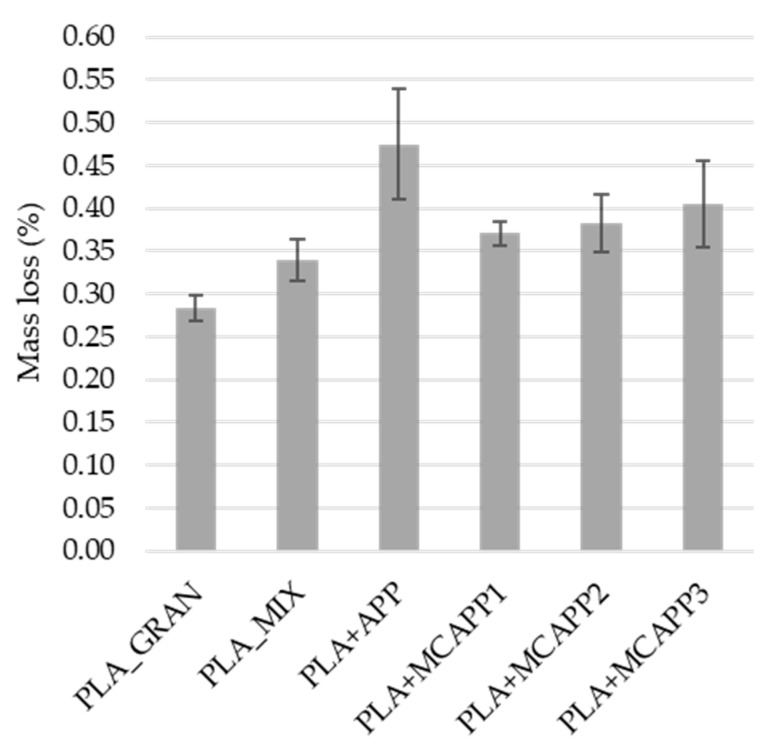
The average mass loss of the samples after water soaking test.

The mass loss of the flame retarded biocomposite sample after the water soaking test can be seen in Figure 12. All the samples lost some weight during the process, likely due to abrasion and dissolution of APP. It can be seen that the PLA + APP lost the most mass during the soaking process, 67% and 40% more than the neat PLA samples. In contrast, the mass loss of the microencapsulated additives is noticeably lower. Even with the thinnest bioresin shell (MCAPP1), the mass loss is only 0.37% greater than that of the additive-free PLA_MIX sample.

Analysis of variance (ANOVA) was performed with Statistica (TIBCO Software Inc. 3307 Palo Alto, CA, USA) to evaluate the effect of water treatment. The results indicated that the type of the additive had a significant influence on the measured mass loss (*p*-value = 0.0000, significance level: 5%). Making post-hoc investigations with Fisher’s Least Significant Difference (LSD) test, it was confirmed with pairwise comparison, that the MCAPP-containing samples significantly differ from the PLA_APP sample, but the difference between the MCAPP samples with different shell thickness is not significant. Based on these results, it was concluded that the microencapsulation with bioepoxy shell effectively improved the water-resistance of APP and thus, the water durability of the flame retarded biocomposites.

#### 2.2.6. Tensile Tests

**Figure 13:**
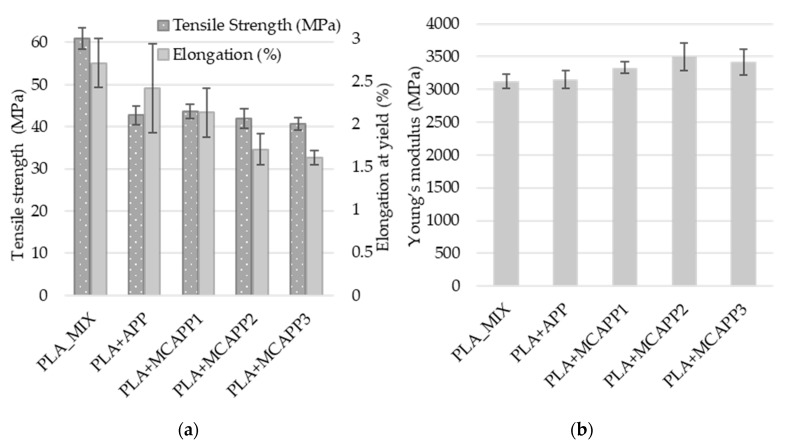
Mechanical properties of the specimens: (**a**) tensile strength and relative elongation at yield, (**b**) Young’s modulus.

Tensile tests were performed to investigate the effect of bioepoxy encapsulation of APP on the mechanical properties of the flame retarded PLA composites. In Figure 13a, the tensile strength and elongation at yield values of the composites are shown, while the measured moduli are plotted in Figure 13b.

APP, both in neat and encapsulated form, acts as a non-reinforcing filler in the polymer matrix. The tensile strength of the flame retarded PLA composites is about 30% lower than that of the additive-free PLA, as expected based on the relatively large particle size and low aspect ratio of the additive. On the other hand, modulus of the PLA composites shows an increasing tendency with the thickness of the bioepoxy shell which is explained by the presence of stiff interphase with properties somewhere between those of PLA and APP and also related to the increased crystalline contents of the MCAPP-containing samples (see Table 4). Analysis of variance (ANOVA) was performed to evaluate the effect of the additives on Young’s modulus. Results indicated that the type of the additive had a significant influence on the measured properties (*p*-value = 0.0031, significance level: 5%). Making post-hoc investigations with the Fisher’s LSD test, it was confirmed with the pairwise comparison that the PLA + MCAPP2 and PLA + MCAPP3 samples significantly differ from both PLA_MIX and PLA+APP samples. In parallel, the rigid filler reduces deformability and thus elongation at yield, which is more pronounced when the APP is encapsulated with bioepoxy resin.

#### 2.2.7. Flame Retardancy Tests

The flame-retardant efficiency of the additives in the PLA matrix was evaluated at 15 wt% loading using the limiting oxygen index (LOI), UL-94 tests, and mass loss type calorimetry (MCC). It has to be noted that 15 wt% of APP by itself is generally insufficient to reach the V-0 rating according to the standard UL-94 test in PLA matrix [44]. With the modification of APP, our aim was to achieve better flame retardant performance in PLA at this relatively low total weight percentage (15 wt%) of additives. The measured LOI values and UL-94 vertical burning classifications, including the average burning times and the results of the cotton ignition tests, are given in Table 4.

The two types of reference PLA samples (PLA_GRAN and PLA_MIX) are easily flammable materials with low LOI values and cannot be classified according to the horizontal UL-94 test. In the case of the PLA_MIX sample, the thermo-mechanical processing even resulted in enhanced flammability. The addition of APP and MCAPPs with different composition (APP to bioresin shell ratio) significantly increased the LOI value of PLA. Comparing the values of the FR loaded samples, there is a slight increase in the case of the PLA + MCAPP2 sample.

The UL-94 rating of the 15 wt% neat APP-containing PLA is V-2 due to the formation of flaming droplets. However, using the microencapsulated additives, V-0 ratings were reached. Even the thinnest examined bioepoxy shell layer (MCAPP1) proved to be effective in eliminating the flaming dripping of the PLA composite and also in reducing the after-flame time.

**Figure 14:**
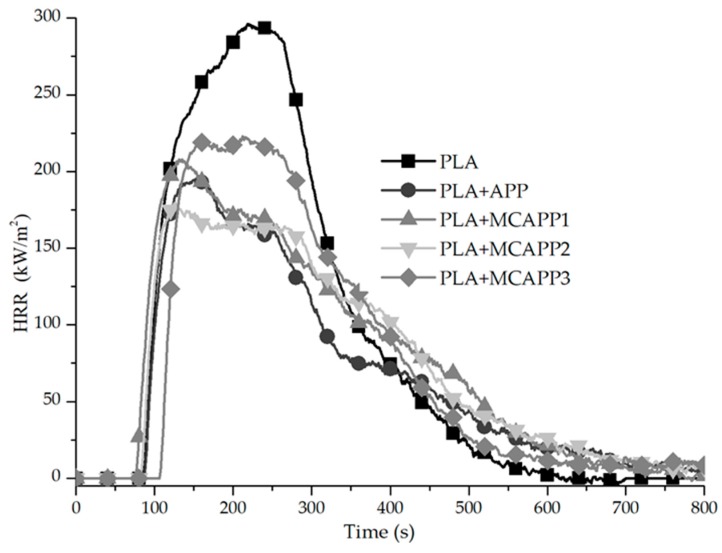
The heat release curves of the biocomposite samples overtime under 35 kW/m^2^ heat flux.

Figure 14 shows the heat release rate curves obtained during mass loss calorimeter tests, while the detailed combustion parameters gained from the mass loss calorimetry measurements are summarized in Table 5.

There was no significant difference in the time to ignition of either of the samples; however, a meaningful change can be observed in the total amount of heat release (THR) and the peak of heat release rate (pHRR) values of the APP-containing samples. One can see from Figure 14, that the pHRR value of the reference PLA_MIX sample is 288 kW/m^2^ and the presence of 15 wt% of neat APP decreased the pHRR to 189 kW/m^2^, which means a 34% reduction. By adding MCAPPs at the same loading, even lower pHRR values were achieved. All the MCAPPs have better fire retardant efficiency than neat APP in PLA matrix, and the MCAPP2 additive is the most effective among the three additives, with the average of 154 kW/m^2^ pHRR, which is 19% lower than that of the untreated APP-containing composite and 46% lower than that of the pure PLA.

Similarly to the pHRR results, the MCAPPs successfully reduced the total heat release (THR) too, from 62.1 MJ/m^2^ of the neat PLA_MIX sample to 42.1, 38.5, and 42.4 MJ/m^2^ in the order of shell thickness (which corresponds to a 32.0%, 37.9%, and 31.5% decrease), respectively. Considering these results, the MCAPP2 additive was found to be the most effective by providing the lowest THR, 37.9% lower compared to the neat polymer and 13.3% lower than the heat emission of the untreated APP-containing PLA sample.

**Figure 15:**
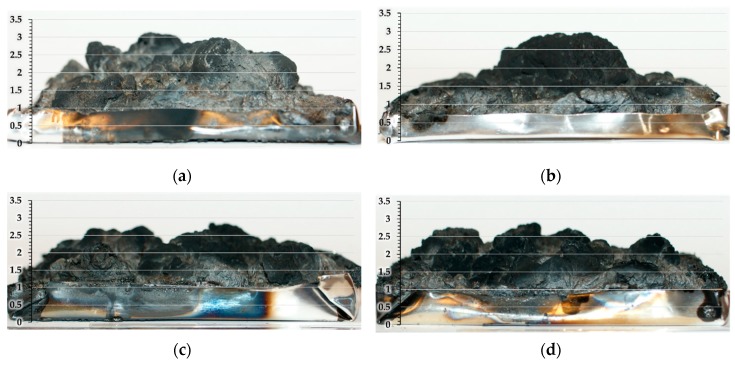
The char residues after mass loss calorimetry of: (**a**) PLA + APP, (**b**) PLA + MCAPP1, (**c**) PLA + MCAPP2, and (**d**) PLA + MCAPP3.

**Figure 16:**
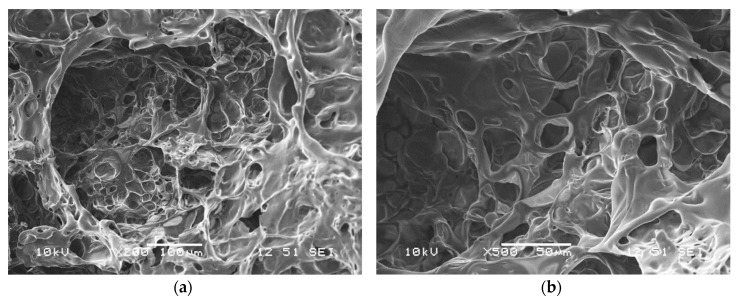
The formed pore structure of the char residue observed by SEM imaging: (**a**) 200× magnification, (**b**) 500× magnification.

Formation of an expanded charred layer, with an uneven surface and a height of about 1–3 cm, was observed for all the additive-containing samples (Figure 15). The residues were also analyzed using SEM, the typical microstructure of the intumescent chars are shown in Figure 16. All the expanded chars showed flexible character with mainly closed cells. The formation of such a char structure can effectively prevent the heat transfer between the flame zone and the burning substrate and thus protect the underlying materials from further combustion [45].

Table 5 shows the average residual mass obtained after the mass loss calorimetric measurements: from the PLA + APP 26.6% residue remained and from the MCAPP samples, 28.0%, 28.8%, and 26.2% remained, respectively. It can be concluded that the bioepoxy shell, as an available carbonizing component, is effective in increasing the flame retardant efficiency of APP, especially considering that the bio-resin was added at the expense of APP to keep constant loading percentage in the PLA matrix.

Although based on the TGA analyses the best char promoting behavior was found for the MCAPP3 additive with the thickest bioresin shell (Table 6), the better flame retardant performance was evinced for the MCAPP2 during all the performed flammability tests. This observation is likely connected with the different dispersion of the MCAPPs in the PLA matrix, as found during SEM observation (Figure 8). The bioepoxy shell is an effective bio-based charring agent, but it can only be effectively utilized when adequate dispersion is achieved. The inhomogeneous distribution of the MCAPP3 particles is assumed to be highly responsible for the deterioration of the flammability and mechanical properties of the PLA + MCAPP3 composite compared to the PLA composites flame retarded with encapsulated APP particles with thinner bioepoxy shell.

## 3. Materials and Methods

### 3.1. Materials

**Figure 17:**
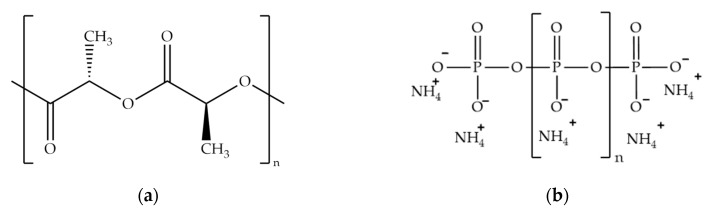

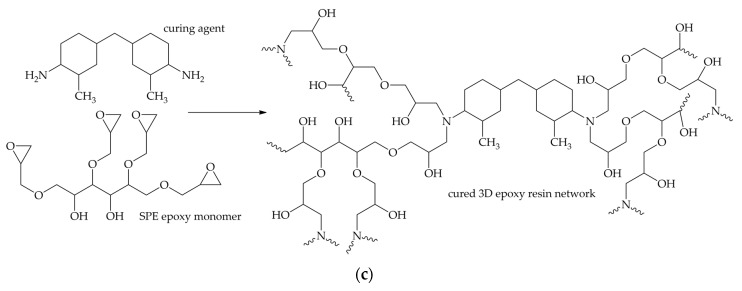
The structure of: (**a**) l-polylactic acid, (**b**) ammonium-polyphosphate, and (**c**) sorbitol polyglycidyl ether and Ipox MH 3122 (2,2′-dimethyl-4,4′-methylenebis(cyclohexylamine)) cross-linking agent and the structure of the cured epoxy resin network.

Ingeo™ Biopolymer 4032D-type extrusion grade PLA, supplied by NatureWorks LLC (Minnetonka, MN, USA), was used as polymer matrix material. It contains mainly polylactic acid of l configuration, but according to its datasheet, it also contains 0.2% residual monomer, and also 1.5% of d-isomer. Exolit^®^ AP 422-type ammonium-polyphosphate, received from Clariant (Clariant AG, Muttenz, Switzerland), was applied in neat and encapsulated form as a flame retardant. For the preparation of bioresin shells, sorbitol polyglycidyl ether (SPE, epoxide equivalent weight 160–195 g/eq, ERISYS^®^ GE-60) purchased from Emerald Performance Materials LLC (Vancouver, WA, USA) bio-based epoxy component was combined with Ipox MH 3122 (Ipox Chemicals Kft., Budapest, Hungary) (2,2′-dimethyl-4,4′-methylenebis(cyclohexylamine)) cycloaliphatic amine-type cross-linking agent. Absolute ethanol was purchased from Merck (Merck KGaA, Darmstadt, Germany). The structures of the aforementioned materials can be seen in Figure 17.

### 3.2. Preparation of Microencapsulated Ammonium-Polyphosphate (APP)

The sorbitol polyglycidyl ether (SPE) bioepoxy component (4, 8, and 12 g, representing 1:10, 2:10, and 3:10 mass ratio of SPE to APP, respectively) and the cyclic amine-type cross-linking agent (33 wt% of the SPE) were dispersed in 50 mL absolute ethanol and stirred until the resin was dispersed in the solvent. Then, pure APP (40 g) was added into the mixture with continuous stirring. Then, it was refluxed at the boiling point of the ethanol (at 78 °C) for 4 h. Then, the mixture was cooled to room temperature, filtered, washed with absolute ethanol, and dried (and post-cured) at 120 °C for 48 h. The clumped particles were powdered in a hand mortar and finally, the microencapsulated ammonium-polyphosphate additives (MCAPP) were obtained. Table 6 shows the theoretical composition of the prepared tree types of MCAPP additives differing in the thickness of the bioepoxy resin shell layers.

### 3.3. Preparation of Flame Retarded PLA Composites

#### 3.3.1. Kneading

The flame retarded PLA composites were prepared using a HAAKE™ Rheomix OS Lab Mixer-type internal mixer (Haake Technik GmbH, Vreden, Germany) in 200 g batches. The previously dried PLA granules were melted at 185 °C and then mixed with the dried APP-based additives (15 wt% each) for 15 min with a rotor speed of 50 min^−1^. In Table 7, the compositions of the prepared flame retarded PLA composites are shown.

#### 3.3.2. Moulding

The kneaded materials were dried overnight at 70 °C and then hot-pressed using a Fontjine LabEcon300 Junior heated platen press (Fontijne Grotnes Inc, Niles, MI, USA). About 40 g of each mixed sample was heated to 185 °C in a mold of 100 × 100 × 3 mm^3^ size, then pressed under 20 kN (2.048 MPa) for 2 min, then under 40 kN (4.096 MPa) for 8 min, and finally cooled to 50 °C under 40 kN (4.096 MPa). The specimens for flammability and mechanical testing were obtained by cutting the plates with a bandsaw.

### 3.4. Characterization Methods

#### 3.4.1. Fourier Transform Infrared Spectroscopy

Infrared spectra (4000–400 cm^−1^) of the microcapsules were recorded using a Bruker Tensor 37-type Fourier transform infrared (FTIR) spectrometer (Bruker Corporation, Billerica, MA, USA) equipped with deuterated triglycine sulfate (DTGS) detector with a resolution of 4 cm^−1^. Before testing, the powder of the microcapsules was mixed with potassium bromide (KBr) powder and cold-pressed into a suitable disk for FTIR measurement.

#### 3.4.2. Raman Spectroscopy

Raman mapping was carried out using a Horiba Jobin–Yvon LabRAM (Longjumeau, France) system coupled with an external 532 nm frequency-doubled Nd:YAG (neodymium-doped yttrium aluminium garnet; Nd:Y_3_Al_5_O_12_) laser source and an Olympus BX-40 optical microscope. The surface of a microencapsulated particle was mapped with an objective of 100× magnification (laser spot size: 0.7 µm). The measured area was approximately 30 µm by 30 µm, with 1 µm step size in both X and Y dimensions. The component concentrations were estimated with the classical least squares (CLS) method using the reference spectra of the pure components collected on the same device under the same conditions. Visualized score maps were created with LabSpec 5.41 (Horiba Jobin–Yvon). The spectrograph was set to provide a spectral range of 100–3400 cm^−1^ and 2 cm^−1^ resolution. The acquisition time of a single spectrum was 5 s, and three spectra were averaged at each measured point.

#### 3.4.3. Scanning Electron Microscopy

Scanning electron microscopic (SEM) micrographs of the microcapsules and the cryogenic fracture surface of the flame retarded PLA samples were taken using a JEOL JSM-5500 LV type apparatus (JEOL Ltd., Akishima, Tokyo, Japan) at an accelerating voltage of 10 keV. Before the examination, all the samples were sputter-coated with a conductive gold layer in order to prevent charge build-up on the surface.

#### 3.4.4. Calculation of Particle Size Distribution

**Figure 18:**
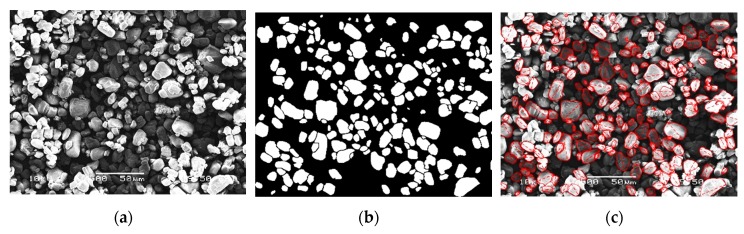
The steps of the particle size distribution measurement: (**a**) The SEM image, (**b**) area of selected particles in the binary image, and (**c**) the selected circumference of the particles and their maximum diameter.

Particle size distribution was determined by image processing of SEM micrographs with 500× magnification (Figure 18). The image processing was carried out with MATLAB’s (The MathWorks, Inc., Natick, MA, USA) Image Processing Toolbox. In the SEM images, the outline of the identified particles were selected (at least 100 for each type of particles), from which the values were calculated. The program calculated the area of the particles, from this area the diameter of a circle of equal projection area (d_EC_) was calculated, which is the diameter of a circle that has the same area as the particle. Then, the maximum diameter (d_M_) was measured, that is the longest distance between any two points along the selection boundary.

#### 3.4.5. Differential Scanning Calorimetry

Differential scanning calorimetry (DSC) measurements were carried out using a Mettler Toledo DSC 3+ (Mettler-Toledo International Inc., Columbus, Ohio, USA) DSC and monitored with STAR^e^ Evaluation Software. Heating-cooling-heating cycle experiments were performed under 25 mL/min nitrogen gas flow, covering a temperature range of 25–200 °C with a heating rate of 10 °C/min and a cooling rate of 2 °C/min, respectively. About 10 mg of sample was used in each test. The degree of crystallinity (χc) of the samples was calculated according to Equation (1), where ΔH_m_ indicates the melting enthalpy, ΔHc is the cold crystallization enthalpy, ΔH_m0_ is the melting enthalpy of the 100% crystalline PLA equal to 93 J/g [46], and φ is the weight fraction of the additives.

(1) $$\chi_{c}\left(\%\right)\;=\;\frac{\Delta H_{m}-\Delta H_{c}}{\left(1-\phi \right)\Delta H_{m}^{0}}\times 100\%$$

#### 3.4.6. Thermogravimetric Analysis

Thermogravimetric analysis (TGA) measurements were carried out using a TA Discovery Apparatus (TA Instruments LLC, New Castle, NH, USA) under 100 mL/min nitrogen gas flow. Samples of about 10 mg were positioned in open alumina pans with gold foil and submitted to an isotherm at 50 °C for 10 min, then followed by a heating ramp of 10 °C/min up to 800 °C (the precision on the temperature measurements is ±1.5 °C in the temperature range of 50–800 °C). Interactions between the compounds of the microcapsules can be revealed by comparing the experimental TG curve with a “theoretical” TG curve (*W_theo_*), calculated as a linear combination of the TG curves of the capsule ingredients weighted by their contents, as in Fontaine et al. [47].

(2) $$W_{theo}\left(T\right)\;=\;\sum_{i=1}^{n}x_{i}W_{i}\left(T\right),$$

where *x_i_* is the content of compound “*i*” and *W_i_* is the TG curve of the compound “*i*”. To determine the potential interactions between the two components and their further effects on the thermal stability of the systems, the curves of weight differences between experimental and theoretical TG curves were computed as follows:

(3) $$\Delta W\left(T\right)\;=\;W_{exp}\left(T\right)-W_{theo}\left(T\right),$$

where Δ*W*(*T*) is the curve of weight difference and *W_exp_*(*T*) is the experimental TG curve of the formulation.

#### 3.4.7. Thermogravimetry-FTIR Spectrometry

The thermal behaviour and the relating evolved gaseous decomposition products of the additives were assessed by thermogravimetry-FTIR (TG-FTIR) spectrometry, using a TA Instruments (New Castle, NH, USA) Q5000 apparatus coupled to a Bruker Tensor 37 FTIR (Bruker Corporation, Billerica, MA, USA) machine. A heating rate of 5 °C/min was applied within the temperature range of 25 to 500 °C under ch atmosphere. Resolution in FTIR was set at 4 cm^−1^, spectrum scan frequency at 12 times per minute, and the spectral region at 4000–650 cm^−1^.

#### 3.4.8. Gel Permeation Chromatography

To determine the number-average molecular mass (Mn) of the neat and flame retarded PLA samples, gel permeation chromatography (GPC) measurements were carried out in tetrahydrofuran (THF) at 40 °C, with a flow rate of 1 mL/min and a polymer concentration of 2 μg/mL. The solutions were filtered with a 0.5 µm pore size filter. The measurements were performed with a Waters system (Separation Module Waters e2695, Milford, MA, USA) equipped with three columns (Styragel HR1, Styragel HR3, and Styragel HR4) placed in series and followed by a refractive index (RI) Wyatt detector (WYATT Optilab T-Rex, Santa Barbara, CA, USA). To get the correct mass values for PLA, the experimental values obtained from the GPC traces using polystyrene standards were multiplied by 0.58 [48].

#### 3.4.9. Water-Resistance of Flame Retarded (FR) PLA Composites

18 samples with 100 × 10 × 3 mm^3^ dimensions from each composite were soaked in distilled water for 96 h at 40 °C, the water was changed every 24 h. After the soaking, the samples were dried until a constant weight was achieved for 60 h at 70 °C. The mass of the dried samples was measured before and after the water soaking.

#### 3.4.10. Tensile Tests

Comparative tensile tests were performed on rectangular specimens of 100 × 10 × 3 mm^3^ (width × length × depth) (the gauge length was 70 mm) using a Zwick Z020 universal testing machine (Zwick GmbH and Co. KG, Ulm, Germany) with a crosshead speed of 5 mm/min. 5 specimens were tested from each composite sample. Based on the measured geometric data and the resulting stress-strain curves, the tensile strength (σ_M_), Young’s modulus (E), and the relative elongation at maximum force were calculated for each specimen using the ISO 527-1:2012 standard.

#### 3.4.11. Limiting Oxygen Index

Limiting oxygen index (LOI) was determined on specimens with 100 × 10 × 3 mm^3^ dimensions according to ISO 4589 standard using an apparatus made by Fire Testing Technology Ltd. (East Grinstead, West Sussex, UK). 3 specimens were tested in all cases.

#### 3.4.12. UL-94

Standard UL-94 flammability tests (according to Standard for Safety of Flammability of Plastic Materials for Parts in Devices and Appliances testing, from Underwriters Laboratories (UL LCC, Northbrook, IL, USA)) were performed in a Fire Testing Technology (East Grinstead, West Sussex, UK) UL 94 Chamber (the device has a stopwatch that accurately measures tenths of a second) according to ISO 9772 and ISO 9773, the specimen dimensions for the test were 100 × 10 × 3 mm^3^. 5 specimens were tested in all cases.

#### 3.4.13. Mass Loss Calorimetry

Mass loss-type calorimeter tests were carried out by an instrument delivered by Fire Testing Technology Ltd., (East Grinstead, West Sussex, UK), using the ISO 13927 standard method. Specimens (100 × 100 × 3 mm^3^) were exposed to a constant heat flux of 35 kW/m^2^ simulating a mild fire scenario. The ignition was provided by a spark plug located 13 mm above the sample. The main characteristic of fire properties, including heat release rate (HRR) as a function of time, time to ignition (TTI), and total heat release (THR), were determined. When measured at 35 kW/m^2^, HRR and THR values are reproducible to within ±10%. The data reported in this article are the worst of the three replicated experiments.

## 4. Conclusions

Sorbitol-based epoxy resin was found to be an effective bio-based charring agent and successfully applied on the surface of APP particles to create a complex (3 in 1) intumescent flame retardant additive for PLA. Besides the noticeable improvement in the flame retardant properties, the encapsulation of APP with bioepoxy resin provides a better filler–matrix interaction, increased modulus, and improved water-resistance to the PLA composites. It is proposed that other types of bio-based epoxy resins (such as sugar-based epoxy resins) could also be utilized in other polymer types/systems to provide a more effective and green fire retardancy solution.

## Figures and Tables

**Table 1:** Equivalent circle diameter and the maximum diameter of the particles.

Computed Parameters	APP	MCAPP1	MCAPP2	MCAPP3
Average d_EC_ (µm)	9.0 ± 3.9	10.1 ± 4.7	11.1 ± 5.1	15.7 ± 6.1
Average d_M_ (µm)	11.5 ± 5.1	12.8 ± 6.2	13.5 ± 6.4	19.4 ± 7.4

**Table 2:** Thermal properties of the PLA composites as measured by heating/cooling/heating differential scanning calorimetry (DSC) experiments.

Thermal Properties	Symbol and unit	PLA MIX	PLA + APP	PLA + MCAPP1	PLA + MCAPP2	PLA + MCAPP3
Recrystallisation enthalpy ^1^	ΔH_m_ (J/g)	4.27	3.47	3.47	3.30	4.24
Melting enthalpy	ΔH_m_ (J/g)	44.82	38.63	39.92	40.48	43.10
Crystallinity ^1^	(%)	51.3	44.5	46.1	47.0	49.2
Cooling crystallization heat ^2^	ΔH_c_ (J/g)	2.91	34.02	37.69	39.14	40.13
Cooling crystallization peak ^2^	T_c_ (°C)	109.7	105.6	112.4	116.6	112.4
Melting enthalpy ^3^	ΔH_m_ (J/g)	13.60	38.65	42.23	43.05	41.59
Crystallinity ^3^	(%)	17.1	48.5	53.0	54.1	52.2

^1^ denotes the first DSC run (heating); ^2^ denotes second DSC run (cooling), ^3^ denotes third DSC run (heating) in heat/cool/heat DSC procedure, respectively.

**Table 3:** The TGA data of the samples.

Formulation	T_max_ (°C) ^1^	T_5%_ (°C) ^2^	Weight at 800 °C (%)	Mass loss rate at T_max_ (%/°C)
PLA_GRAN	360	325	0.0	3.0
PLA_MIX	365	327	0.0	3.4
PLA + APP	363	327	4.7	2.4
PLA + MCAPP1	363	322	5.8	2.4
PLA + MCAPP2	366	315	6.3	2.2
PLA + MCAPP3	368	310	8.4	2.0

^1^ The temperature corresponding to maximum-rate decomposition. ^2^ The temperature corresponding to a 5% weight loss.

**Table 4:** The results of the limiting oxygen index (LOI) and UL-94 tests on the reference and flame retarded biocomposite samples.

Sample	LOI (%)	UL-94 (3 mm)		
		t_1_/t_2_ (s) ^1^	Cotton Ignition	Rating
PLA_GRAN	21.5	-/-	yes	N.R. ^2^
PLA_MIX	20.5	11/-	yes	N.R. ^2^
PLA + APP	28.0	4.8/1.4	yes	V-2
PLA + MCAPP1	28.5	1.3/1.4	no	V-0
PLA + MCAPP2	29.0	2.8/0.9	no	V-0
PLA + MCAPP3	28.0	2.2/0.6	no	V-0

^1^ represent the average after-flame time after the flame application of first and second 10 s, and “-” means complete combustion for samples. ^2^ No rating.

**Table 5:** The average ignition time, the peak of heat release rate (pHRR), total heat release (THR), and average residual mass for the samples.

Formulation	t_ign_ (s)	pHRR (kW/m^2^)	pHRR Decrease * (%)	THR (MJ/m^2^)	THR decrease * (%)	Residue (wt%)
PLA GRAN	82 ± 2	284 ± 7	1.4	61.9 ± 1.7	–0.3	1.7 ± 0.4
PLA MIX	87 ± 2	288 ± 18	0	62.1 ± 1.2	0	1.4 ± 0.2
PLA + APP	82 ± 4	189 ± 13	34.2	44.4 ± 2.0	28.2	26.6 ± 3.0
PLA + MCAPP1	82 ± 3	175 ± 14	38.9	42.1 ± 3.8	32.0	28.0 ± 4.3
PLA + MCAPP2	80 ± 3	154 ± 8	46.2	38.5 ± 3.5	37.9	28.8 ± 2.8
PLA + MCAPP3	84 ± 4	167 ± 17	41.7	42.4 ± 2.2	31.5	26.2 ± 4.7

* compared to PLA_MIX.

**Table 6:** Theoretical composition of the microcapsules.

Sample Code	APP (wt%)	SPE (wt%)	Curing Agent (wt%)
MCAPP1	88.24	8.83	2.93
MCAPP2	78.90	15.80	5.30
MCAPP3	71.43	21.43	7.14

**Table 7:** Formulations of the PLA composites.

Sample code	PLA (wt%)	MCAPP (wt%)	
		APP (wt%)	SPE Bioresin (wt%)
PLA_GRAN	100	0	0
PLA_MIX	100	0	0
PLA + APP	85	15	0
PLA + MCAPP1	85	13.4	1.6
PLA + MCAPP2	85	11.8	3.2
PLA + MCAPP3	85	10.7	4.3
